# Reply to Jones et al.: Opportunities and considerations for valuing seagrass ecosystem services

**DOI:** 10.1073/pnas.2500092122

**Published:** 2025-02-18

**Authors:** Joleah B. Lamb, Nur Abu, Steven R. Limbong, Jamaluddin Jompa, William E. Feeney, C. Drew Harvell

**Affiliations:** ^a^Department of Ecology and Evolutionary Biology, University of California, Irvine, CA 92697; ^b^Department of Environmental Engineering, Muhammadiyah University of Sorong, Sorong 98416, Indonesia; ^c^Ministry of National Development Planning, Government of Indonesia, Jakarta 10310, Indonesia; ^d^Faculty of Marine and Fisheries Sciences, Hasanuddin University, Makassar 90245, Indonesia; ^e^Doñana Biological Station, Spanish National Research Council, Seville 41092, Spain; ^f^Department of Ecology and Evolutionary Biology, Cornell University, Ithaca, NY 14853

As the global distribution of seaweed farming expands (1), seagrass ecosystems face a high risk of removal in coastal areas where they coincide. Valuing ecosystem services is an informed approach for addressing imminent challenges associated with sustainable development (2). Fiorenza et al. (3) use a natural experiment to build upon a growing body of evidence that seagrass ecosystems across tropical and temperate regions reduce marine bacterial pathogens and disease (4–6) and extend this to estimate that conserving seagrass ecosystems within seaweed cultivation areas could reduce revenue loss per harvest via the mitigation of a globally widespread seaweed disease. Importantly, Jones et al. (7) highlight the complexity of ecological, economic, and social considerations that are required to inform decision-making prior to implementation and advise that scaling is premature. We agree and recognize that our study provides an analysis of a single economic dimension of value for a select number of beneficiaries, while also appreciating that determining the full range of services an ecosystem provides can limit conservation efforts (8). Although Fiorenza et al. (3) present the global scale relevance at which co-cultivation conditions exist, we do emphasize that scaling marine farming production can come at the expense of coastal ecosystems (9, 10) and acknowledge that a number of challenges still exist in optimizing co-cultivation practices. To ensure that informed and equitable outcomes are achieved (11), we support the viewpoint that further nuanced discussions are required.
